# Nurses' Food and Feeding

**Published:** 1886-11-06

**Authors:** 


### NURSES' FOOD AND FEEDING.

Knowing what an important matter it is that nurses should be well fed, I hope you will grant me space in your valuable paper to point out a serious omission in the dinner-list of the Seamen's Hospital and of "Victoria", namely, that on three days of the week no second course is allowed. Victoria complains of nurses' dinners being indigestible, but surely few dinners could be conceived more so than one she proposes,—"sausages." I think most hospital managers will agree with me that soup or pudding, in addition to meat daily, is true economy, not only as regards the nurses' health, but also in regard to the butcher's bill.

P. W. K.

County Infirmary, Oct. 29th, 1886.

